# A mutation in the cytosolic O-acetylserine (thiol) lyase induces a genome-dependent early leaf death phenotype in *Arabidopsis*

**DOI:** 10.1186/1471-2229-10-80

**Published:** 2010-04-29

**Authors:** Reza Shirzadian-Khorramabad, Hai-Chun Jing, Gerja E Everts, Jos HM Schippers, Jacques Hille, Paul P Dijkwel

**Affiliations:** 1 Molecular Biology of Plants, Groningen Biomolecular Sciences and Biotechnology Institute, University of Groningen, Kerklaan 30, 9751 NN, Haren, The Netherlands; 2 Centre for Bioenergy Plants Research and Development, Institute of Botany, Chinese Academy of Sciences, Beijing, 100093, China; 3 Institute of Molecular BioSciences (IMBS), Massey University, Private Bag 11222, Palmerston North, New Zealand; 4 University of Guilan, Department of Agronomy and Plant Breeding, P.O. Box 41635-1314, Rasht, Iran; 5 University of Potsdam, Department of Molecular Biology, Karl-Liebknecht-Str. 24-25, Haus 20, 14476 Potsdam-Golm, Germany

## Abstract

**Background:**

Cysteine is a component in organic compounds including glutathione that have been implicated in the adaptation of plants to stresses. *O*-acetylserine (thiol) lyase (OAS-TL) catalyses the final step of cysteine biosynthesis. OAS-TL enzyme isoforms are localised in the cytoplasm, the plastids and mitochondria but the contribution of individual OAS-TL isoforms to plant sulphur metabolism has not yet been fully clarified.

**Results:**

The seedling lethal phenotype of the *Arabidopsis onset of leaf death3-1 *(*old3-1*) mutant is due to a point mutation in the *OAS-A1 *gene, encoding the cytosolic OAS-TL. The mutation causes a single amino acid substitution from Gly^162 ^to Glu^162^, abolishing old3-1 OAS-TL activity *in vitro*. The *old3-1 *mutation segregates as a monogenic semi-dominant trait when backcrossed to its wild type accession Landsberg *erecta *(L*er*-0) and the Di-2 accession. Consistent with its semi-dominant behaviour, wild type L*er*-0 plants transformed with the mutated *old3-1 *gene, displayed the early leaf death phenotype. However, the *old3-1 *mutation segregates in an 11:4:1 (wild type: semi-dominant: mutant) ratio when backcrossed to the Colombia-0 and Wassilewskija accessions. Thus, the early leaf death phenotype depends on two semi-dominant loci. The second locus that determines the *old3-1 *early leaf death phenotype is referred to as *odd-ler *(for *old3 *determinant in the L*er *accession) and is located on chromosome 3. The early leaf death phenotype is temperature dependent and is associated with increased expression of defence-response and oxidative-stress marker genes. Independent of the presence of the *odd-ler *gene, *OAS-A1 *is involved in maintaining sulphur and thiol levels and is required for resistance against cadmium stress.

**Conclusions:**

The cytosolic OAS-TL is involved in maintaining organic sulphur levels. The *old3-1 *mutation causes genome-dependent and independent phenotypes and uncovers a novel function for the mutated OAS-TL in cell death regulation.

## Background

The biogeochemical sulphur cycle has a major impact on climate and life. The sulphur-containing amino acid cysteine forms the exclusive building block for organic compounds including glutathione that have been implicated in the adaptation of plants to a wide range of biotic and abiotic stresses [1,2]. Cysteine synthesis creates a link between sulphur reduction and amino acid metabolism and therefore is a point of convergence for nitrogen and sulphur assimilation. Sulphur assimilation starts with the transport of anionic sulphate into plant cells by a family of plasma membrane associated proton/sulphate co-transporter proteins [3]. Through serial enzymatic reactions, sulphate is first converted into sulphide and cysteine biosynthesis is subsequently catalysed by a bienzyme complex called the cysteine synthase complex (CSC). The initial reaction catalyzed by CSC is the formation of O-acetylserine (OAS) from serine and acetyl- CoA by the activity of serine acetyltransferase (SAT) proteins. Subsequently, O-acetylserine(thiol)lyase (OAS-TL) catalyses the incorporation of sulfide into OAS producing cysteine [4,5]. SAT requires the presence of excessive amounts of OAS-TL to gain full activity, whereas OAS-TL activity is low when bound to SAT [6-8]. SAT and OAS-TL enzyme isoforms are distributed within the cell compartments cytoplasm, plastid and mitochondria and the CSC and its subcellular compartmentation have been suggested to play a crucial role in the control of cysteine biosynthesis [9,1]. In *Arabidopsis thaliana*, five and nine genes encode for SAT- and OAS-TL-like proteins, respectively [9]. The SAT proteins show functional redundancy *in vivo *and *Arabidopsis *plants with mutations in any four of the five SAT genes survived although three of the quadruple mutants showed dwarfism [10]. Four genes encoding OAS-TLs are strongly transcribed according to the Genevestigator database [11], the *Arabidopsis *e-FP browser database [12] and individual studies [13-15]. These genes encode cytoplasmic (*OAS-A1*), plastidic (*OAS-TL C*) and mitochondrial (*OAS-TL B *and *CYSC1*) isoforms [15,16]. OAS-A1 is probably the only functional cytosolic OAS-TL in *Arabidopsis *and is responsible for a major part of the total OAS-TL activity in the cell [17,1,18]. The contribution of each OAS-TL isoform to plant sulphur metabolism has not yet been fully clarified. Knock out of the cytosolic OAS-TL isoform reduces total cellular OAS-TL activity by 44 to 80%, however no apparent phenotypic differences were observed between the mutant and the wild type when grown under non-stressed conditions [17,18]. Nevertheless, the antioxidant capacity of the cytosol was perturbed [17]. Cysteine is also found to be the major factor controlling glutathione (GSH) biosynthesis and phytochelatins (PCs) [19-21]. GSH and other secondary organic sulphur compounds are involved in the scavenging of free radicals and hence have been implicated in the adaptation of plants to a wide range of stresses including the detoxification of xenobiotics and the protection against heavy metal toxicity [22,23,2].

Here we report that the lethal *old3-1 *phenotype is the consequence of a point mutation in the gene encoding the cytosolic OAS-TL. The *old3-1 *phenotype is associated with elevated expression of defence-response and oxidative stress marker genes. Interestingly, the semi-dominant phenotype caused by the mutated protein is depending on genomic context resulting in an early onset of leaf death in L*er*-0 and Di-2, but not in the Col-0 and Ws-0 accessions. Since the mutated protein has no OAS-TL activity *in vitro*, these data suggest a novel genome-dependent function of the mutated cytosolic OAS-TL.

## Results

### The *old3-1 *early leaf death phenotype is a temperature-dependent trait

**Figure 1:**
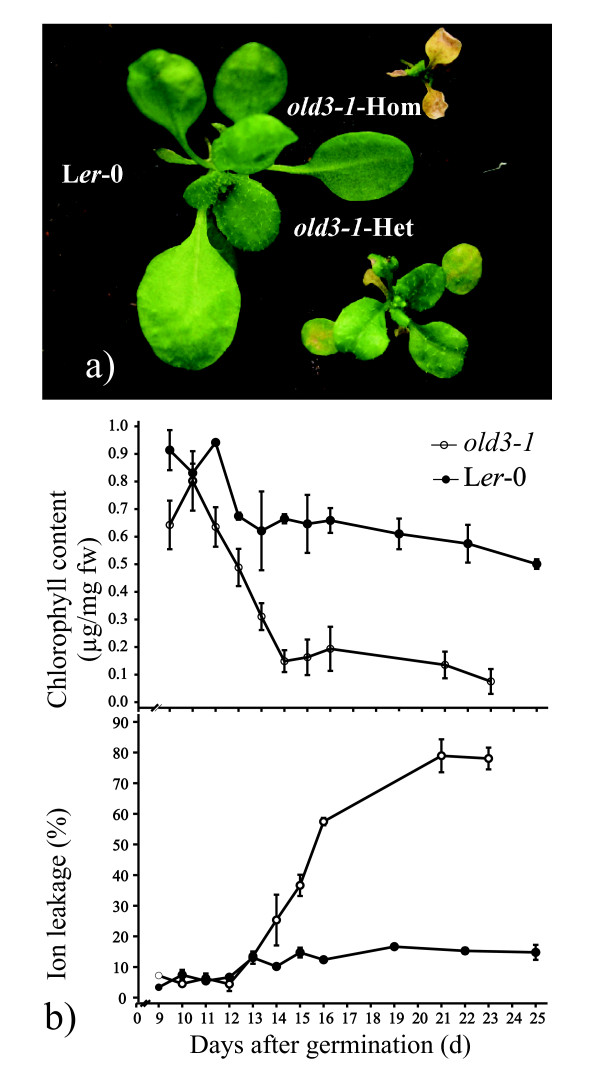
**The early leaf death phenotype in *old3-1 *mutant plants**. a) Representative 21-day-old soil-grown wild type (L*er*-0), *old3-1 *homozygous and *old3-1 *heterozygous plants grown in standard conditions at 21°C. b) Chlorophyll content and ion leakage of cotyledons from L*er*-0 and *old3-1 *homozygous plants grown for up to 25 days. Each data point is shown as mean ± sd from 4 replicates.

**Figure 2:**
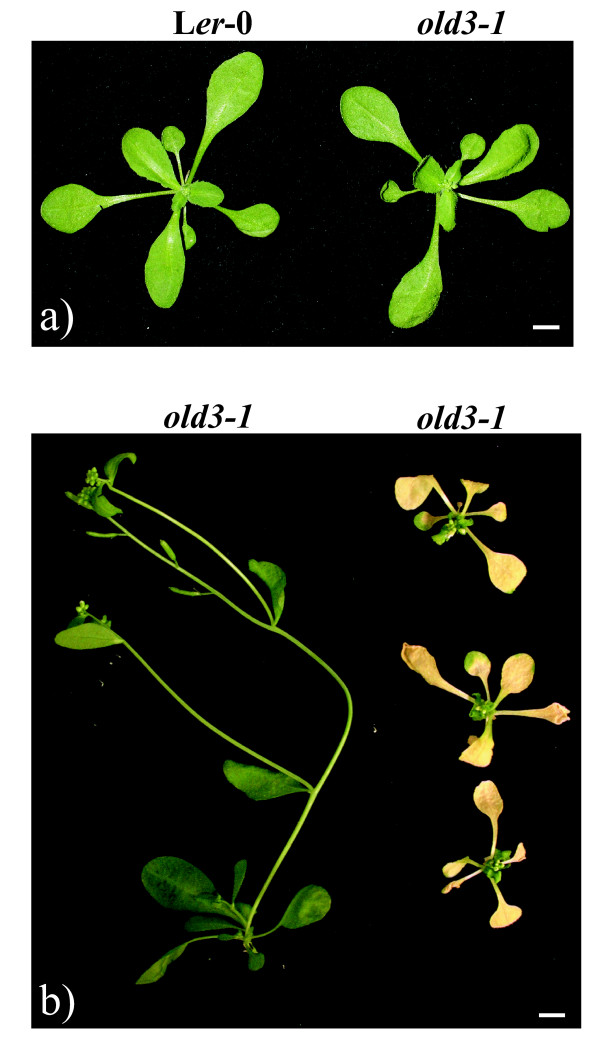
**Temperature-dependent phenotypes of *old3-1 *plants**. a) Wild type (L*er*-0) and homozygous 21-day-old *old3-1 *plants grown at 28°C. b) Representative *old3-1 *mutants were soil-grown either in 50 μmolm^- 2^s^-1 ^cool white fluorescent light and 28°C for 32 days (left) or in 50 μmolm^- 2^s^-1 ^cool white fluorescent light and 28°C for 16 days, and then for another 16 days in 65 μmolm^- 2^s^-1 ^cool white fluorescent light and 21°C (right). Bar represents 0.5 cm.

**Figure 3:**
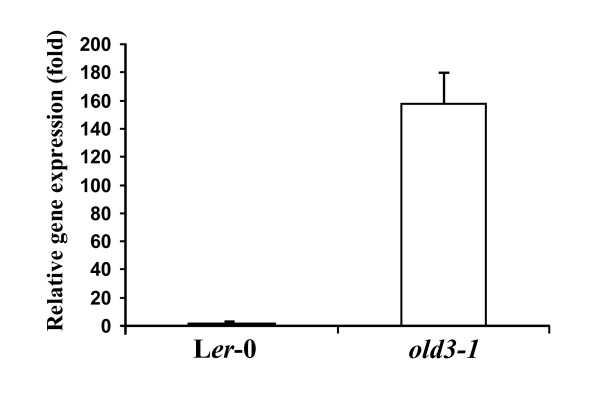
**Relative transcript abundance of the *DEFL *gene**. Relative transcript levels of the general oxidative stress-related marker gene *DEFL *in 12-day-old *old3-1 *and L*er*-0 plants were measured by quantitative Real-Time PCR, using *ACTIN2 *as internal control. The values shown are the means of three repeats ± sd.

The *onset of leaf death3-1 *(*old3-1*) mutant was isolated from an EMS-mutagenised *Arabidopsis *Landsberg *erecta *(L*er*-0) population as a semi-dominant trait [24]. Figure 1a shows that homozygous and heterozygous *old3-1 *plants suffer from an early leaf death syndrome. The first signs of leaf yellowing occur after 12 days of growth in homozygous *old3-1 *seedlings. The visible leaf yellowing was associated with a rapid decrease in chlorophyll content and a concurrent increase in ion leakage (Figure 1b), followed by death of the plant after approximately 4 weeks. Plants heterozygous for the *old3-1 *mutation show premature leaf yellowing but can, in contrast to the homozygous mutants, finish their life cycle. The early leaf death phenotype was found to be a temperature-dependent trait, and growth at 28°C completely suppresses the leaf death phenotype (Figure 2a). Transfer of *old3 *mutants from 28°C to 21°C after 2, 4 or 6 weeks results in a visible leaf death phenotype within 10 days (Figure 2b), suggesting that the temperature dependency is independent of the developmental stage. We examined expression of several marker genes involved in developmental senescence, defence response, and programmed cell death in *old3-1 *and wild type plants. Increased expression of *PR1*, *SAG14, SAG21 *and *SAG13 *was detected in 16- and 24-day-old *old3-1 *plants, while no signs of *SAG12 *expression were found [24]. Marker genes of *PR-1 *and *SAG13 *are associated with programmed cell death as well as oxidative stress signals [25]. Therefore, the expression of a general oxidative stress marker gene *DEFL *(*AT2G43510*) [26] was monitored in 12-day-old *old3-1 *plants, and found to be ~160-fold up-regulated as compared to wild type plants (Figure 3). The results shown here, together with those published previously [24] suggest that early leaf death in *old3-1 *seedlings does not result from activation of a developmental senescence program, but rather a cell death pathway that is related to the plant immune system and stress responses.

### The *old3-1 *phenotype is a genome-dependent trait in *Arabidopsis thaliana*

F_2 _progeny obtained from a cross between heterozygous *old3-1 *and L*er*-0 plants segregates as a monogenic trait for the *old3-1 *phenotype, while the same phenotype segregates in a different ratio when crossed to Col-0 [24] (Table 1). Whereas in L*er*-0 the *old3 *mutation is caused by a single gene defect, two genes are needed for the same phenotype in Col-0. The second gene was designated as *old3-1 *determinant *(odd)*. The L*er*-0 and Col-0 alleles were called *odd-ler *and *odd-col*, respectively. Different segregation ratios were tested based on the number of plants with a homozygous, a heterozygous and wild type phenotype. The 1:2:13, 1:8:7 and 3:7:8 (homozygous: heterozygous: wild type) segregation ratios were dismissed because of chi-square values of higher than 50. Of the other segregation ratios tested, only the 1:4:11 ratio fitted the data (Table 1). Thus two *old3-1 *and two *odd-ler *alleles need to be present for the homozygous leaf death phenotype to be manifested. The heterozygous phenotype requires either one *old3-1 *and two *odd-ler *alleles or one *odd-ler *and two *old3-1 *alleles (Table 2). The presence of the *odd-ler *allele in *old3-1 *plants, rather than the absence of the *odd-col *allele, is required for the manifestation of the *old3-1 *phenotype, thus the *odd-ler *allele is semi-dominant as well. The *old3-1 *heterozygous plants were crossed to two additional accessions. The *old3*-1 phenotype segregated in Di-2 as in L*er*-0, while the Ws-0 accession behaved similar to Col-0 (Table 1). Thus, different *Arabidopsis *backgrounds resulted in two distinct segregation patterns for the *old3-1 *phenotype. The *old3-1 *mutant was crossed to a further 14 different accessions and the F_1 _of 11 of those (Ak-1, Bd-0, Bla-2, Bs-2, Litva, Mt-0, Nok-0, Rubezh, Noe-1, Tsu-1, Wil 2) looked like the F_1 _of the cross with Col-0. The F_1 _of the other 3 crosses had the *old3-1 *heterozygous phenotype (Bu-18, Wa-1, Rsch-0), suggesting the presence of *odd-ler*-like alleles in these accessions.

**Table 1:** Phenotypes and segregation of F_1 _and F_2 _progeny of crosses between heterozygous *old3-1 *plants and various wild type *Arabidopsis *accessions.

		F_1 _phenotype		F_2 _phenotype and segregation^1^				
		
Male	Female	Wild type	*old3-1 *Het	Wild type	*old3-1 *Het	*old3-1 *Hom	χ^2^	Segregation
*old3-1*	L*er*-0	13	12	115	241	123	0.197	1:2:1
Het^1^	Di-2	29	24	183	382	166	0.74	1:2:1

*old3-1*	Col-0	49	0	237	88	19	0.337	1:4:11
Het^1^	Ws-0	53	0	341	119	32	0.18	1:4:11

^1^In the crosses between Het-*old3-1 *and L*er*-0, Di-2 accessions, only the F_1 _progeny with the Het-*old3-1 *phenotypes were selected and used for F_2 _segregation analyses. For the crosses between Het-*old3-1 *and Col-0 and Ws-0, at least ten F_1 _plants were selected and allowed to set seed. The phenotypes of the F_2 _populations were observed and only those in which the *old3-1 *phenotype segregated were used.

**Table 2:** Genotypes and corresponding phenotypes of *old3 *and *odd *allele combinations

Genotype^1^	Phenotype
*OLD3OLD3odd-colodd-col*	Wild type
*OLD3old3-1odd-colodd-col*	Wild type
*old3-1old3-1odd-colodd-col*	Wild type
*OLD3OLD3odd-lerodd-col*	Wild type
*OLD3old3-1odd-lerodd-col*	Wild type
*old3-1old3-1odd-lerodd-col*	Het-*old3-1*
*OLD3OLD3odd-lerodd-ler*	Wild type
*OLD3old3-1odd-lerodd-ler*	Het-*old3-1*
*old3-1 old3-1odd-lerodd-ler*	Hom-*old3-1*

^1^Genotoypes were determined using DNA markers surrounding the *odd *region and a marker that distinguishes between the *OLD3 *and *old3-1 *alleles.

Initial mapping revealed that the *old3-1 *phenotype is linked to a CAPS marker locus called *G4539a *on chromosome 4, and a SSLP marker locus called *K11J14 *on chromosome 3 [24]. Therefore, one of the genome locations carries the *old3-1 *mutation, and the other the *odd-ler *gene. To clarify the position of the *old3-1 *and *odd-ler *loci on the genome, plants heterozygous for the *old3-1 *mutation were crossed to *fca-1 *and *abi1-1 *mutants (both having the L*er*-0 background). The *old3-1 *mutation was found to co-segregate with the *ABI1 *and *FCA *wild type alleles (Data not shown), demonstrating that the *old3-1 *mutation is located on chromosome 4 and the *odd-ler *gene on chromosome 3. Thus, the *old3-1 *early leaf death phenotype is a genome-dependent trait in *Arabidopsis *which requires accession-specific *odd-ler-*like alleles.

### The *OLD3 *gene encodes the cytosolic O-acetylserine (thiol) lyase, OAS-A1

**Figure 4:**
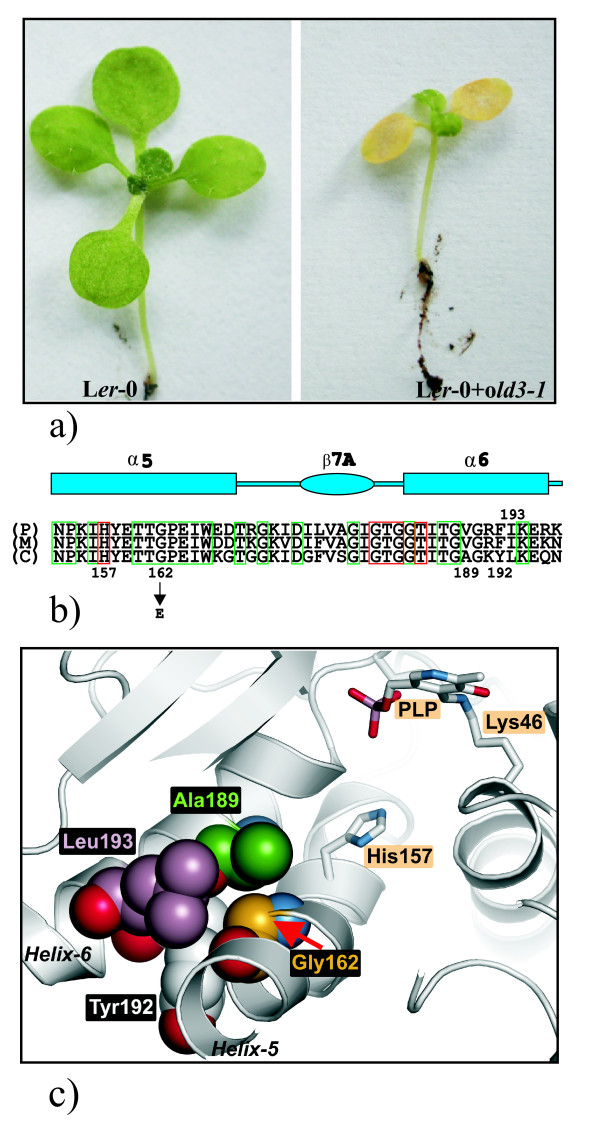
**The *OLD3 *gene encodes the cytosolic OAS-TL**. a) The *old3-1 *genomic region was cloned and transformed into L*er*-0 wild type plants. A 14-day-old wild type L*er*-0 plant and a representative L*er*-0 plant carrying the *old3-1 *transgene are shown. b) Amino acid sequence alignment of the area surrounding G^162 ^of three *Arabidopsis *OAS-TL proteins. The position of the G^162 ^to E^162 ^substitution in the cytosolic OAS-TL is indicated. Amino acids shown in green boxes are highly conserved amino acids; amino acid residues shown in red boxes are involved in the interaction with pyridoxal 5'-phosphate (PLP). The schematic drawing indicates the location of amino acids in helix-5 and helix-6. P (*AT2G43750 *- plastid OAS-TL); M (*AT2G59760 *- mitochondrial OAS-TL); C (*AT4G14880 *- *OAS-A1*). c) The location of PLP, Lys^46^, which is the only residue forming a covalent bond (the Schiff base) with cofactor PLP, and His^157^ that is involved in interacting with PLP are shown by stick drawings (highlighted black on orange). The position of Gly^162 ^(gold) in helix-5 is shown as a CPK model. This residue is in close proximity with residues in helix-6 including Ala^189 ^(green), Tyr^192 ^(white), and Leu^193 ^(purple). Figure made using Pymol, http://www.pymol.org/.

Further mapping placed the *old3-1 *mutation into a 14-kb region on the bacterial artificial chromosome clone FCA2 (accession number Z97337) spanning 8 open reading frames. Sequence analysis revealed a G to A substitution in *AT4G14880 *which encodes the cytosolic *O*-acetylserine (thiol) lyase (OAS-TL), OAS-A1. The mutation results in a Gly^162 ^to Glu^162 ^substitution in the OAS-A1/OLD3 protein. The entire *old3-1 *gene including its promoter region was cloned and transformed into wild-type L*er*-0 plants. Transformation of a single *old3-1 *gene into L*er*-0 is expected to give the *old3-1-het *phenotype. However, because of possible position effects and multiple integrations, a range of expression levels and phenotypes were expected. More then 20 transformants were identified and 15 transformants were tested for the presence of the basta resistance gene, the transgenic *old3-1 *gene and the wild type *OLD3 *gene. All the tested plants had the expected genotype and showed an early leaf death phenotype. However, none of the transformants produced progeny although some phenotypic variation was found with several transformants dying at the two leaf stage similar to the homozygous mutant and others developing up to the flowering stage. Figure 4a shows a photograph of a representative *old3-1 *transformant. Thus, the *old3-1 *mutation results in a mutant protein with an altered or partial function that causes the early leaf death phenotype.

OAS-A1/OLD3 uses pyridoxal 5'-phosphate (PLP) as a cofactor to synthesise cysteine from O-acetylserine (OAS) and sulfide [4,5,27]. According to the amino acid sequence alignment of three OAS-TL isomers in *Arabidopsis*, the *old3-1 *amino acid substitution is located at the fifth α-helix in the middle of a highly conserved area (TTGPEIW) (Figure 4b) [28]. The fifth α-helix, including His^157 ^that interacts with the pyridoxal 5'-phosphate (PLP) binding site, is in close proximity to a mostly conserved region at the sixth α-helix (GAGKYLK) including Ala^189^, Tyr^192 ^and Leu^193 ^(Figure 4c). The Gly^162 ^to Glu^162 ^substitution results in a size and charge difference that may cause a change in the protein structure and activity.

### The old3-1 protein has no *in vitro *OAS-TL activity

**Figure 5:**
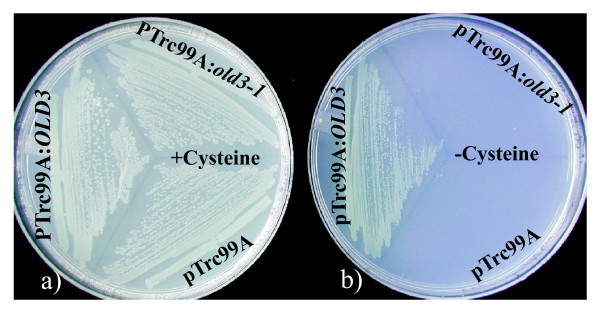
**Genetic complementation of the ΔCys *E.coli *strain NK3**. The empty vector (pTrc99A), and vector harbouring the wild type *OAS-A1 *(pTrc99A:*OLD3*) and mutated *old3-1 *OAS-TL (pTrc99A:*old3-1) *cDNAs were transformed into the ΔCys *E.coli *NK3 strain and plated onto M9 medium with (a) or without (b) 0.5 m*M *cysteine and incubated at 37°C.

The *E.coli Cys- *auxotrophic strain NK3 [29] lacks the cysteine synthase gene and consequently is unable to grow on medium without supplemental cysteine. The OAS-A1/OLD3 is able to complement the *E. coli *NK3 strain [14] and we determined whether the *old3-1 *gene is able to complement the phenotype of the *E.coli *NK3 strain as well. The *old3-1 *and the wild type *OAS-A1/OLD3 *cDNAs were cloned into an expression vector. Subsequently, the *E. coli *NK3 strain was transformed with the expression vectors harbouring the cDNAs and the empty vector and grown on minimal medium M9 with and without cysteine. *E. coli *NK3 transformed with the *OAS-A1/OLD3 *cDNA could grow on medium lacking cysteine, while *E.coli *NK3 carrying the *old3-1 *cDNA and empty vector were unable to grow further (Figure 5). These results confirm that the OAS-A1/OLD3 is able to complement the phenotype of the *E. coli Δcys *NK3 strain and suggest that the cytosolic old3-1 protein has either a reduced or no OAS-TL enzyme activity in *E.coli *NK3.

For further old3-1 OAS-TL activity analysis, the *OAS-A1/OLD3 *and *old3-1 *cDNAs were cloned into the pET15b expression vector. Subsequently the old3-1 and OAS-A1/OLD3 proteins were over-expressed in *E.coli *BL21 (DE3). The proteins were purified using a Ni^2+^-NTA agarose column, and molecular masses on a SDS-PAGE gel were found to be in agreement with the expected molecular mass. We applied 3.1 nM purified OAS-A1/OLD3 and old3-1 OAS-TL proteins to the cysteine precursors OAS and Na_2_S and subsequently OAS-TL activity was measured according to [30]. A specific activity of 84 ± 4 μmol min^-1 ^mg^-1 ^(48 ± 2 s^-1^) was found for the OAS-A1/OLD3, but for the same enzyme concentration no activity was detected for old3-1 OAS-TL. The obtained value for the OAS-A1/OLD3 protein is within those already published (906 ± 39 μmol min^-1 ^mg^-1 ^[6], 2 μmol min^-1 ^mg^-1 ^[31], and 225 μmol min^-1 ^mg^-1 ^[32]). Together, the data show that old3-1 cytosolic OAS-TL has no *in vitro *activity at the enzyme concentrations tested.

### *OAS-A1/OLD3 *affects sulphur balance and thiol levels

**Figure 6:**
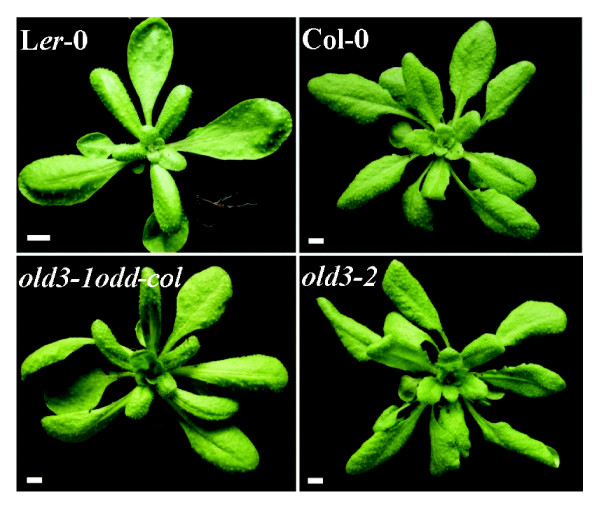
**Phenotypes of wild type and *old3 *mutant plants**. Representative 27-d-old soil-grown plants of L*er*-0, Col-0, *old3-1old3-1odd-col odd-col *(*old3-1odd-col*, carrying the homozygous *old3-1 *mutation and homozygous *odd-col *alleles in an otherwise Col-0/L*er*-0 mixed background) and *old3-2/oas-a1.1 *(*old3-2*, carrying a homozygous T-DNA insertion in the *OLD3 *gene) grown in 250 μmolm^-2^s^-1 ^cool white fluorescent light at 21°C. Bars represent 0.5 cm.

**Figure 7:**
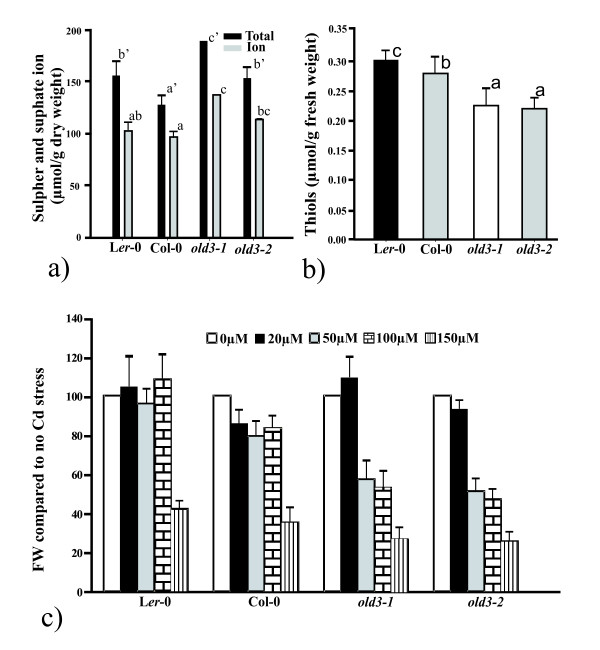
**The function of OAS-A1 on sulphur metabolism and cadmium stress**. a) Total sulphur and sulphate ion levels were measured in dry matter obtained from plants grown for 27 days in soil saturated with 25% Hoagland solution. b) Total water-soluble non-protein sulfhydryl (thiol) compounds were measured in fresh materials obtained from 27-day-old plants. Above-ground parts of 27-day-old plants were harvested for measurement of total sulphur, sulphate ion and thiols content. Data are shown as mean ± sd of four replicates (17 plants for each replicate). Bars with the same letters indicate values that are not significantly different from each other after Duncan multiple variant significance test at a significance level of p < 0.05. c) Effect of CdCl_2 _on fresh weight of *old3-1*, *old3-2 *and their respective wild types. Fresh weight was measured after 16 days of growth on the indicated concentration of CdCl_2_. Fresh weight is expressed as percentage of the fresh weight when grown without CdCl_2 _(set at 100). Data are shown as mean ± sd of three replicates (20 seedlings for each replicate).

Under sulphur-sufficient conditions, metabolites such as cysteine and glutathione act as regulators of sulphur uptake and assimilation [33]. In order to clarify if the mutated cytosolic old3-1 OAS-TL has some effects on availability of down-stream or up-stream compounds in the cysteine biosynthesis pathway, we measured the contents of total sulphur and sulphate ion as direct measurements of plant sulphur balance. Total thiol levels were measured as a parameter of plant sulphur metabolism [34,21,35]. To be able to distinguish between direct effects of the mutated OASTL and indirect effects as a result of the early leaf death phenotype, we isolated the *old3-2 *mutant as described in the Methods section. This mutant carries a homozygous T-DNA insertion in the *OAS-A1/OLD3 *gene and is identical to the *oasa1.1 *mutant as described by Lopez-Martin et al. [17] (results not shown). The *old3-2/oas-a1.1 *mutant has the Col-0 (*odd-col*) background and similar to the *old3-1old3-1odd-colodd-col *line (carrying the homozygous *old3-1 *mutation and *odd-col *alleles in an otherwise Col-0/L*er*-0 mixed background), did not show the early leaf death phenotype (Figure 6). Thus, the lack of OAS-TL activity in the *old3-2/oas-a1.1 *mutants does not cause a leaf death phenotype. Figures 7a and 7b show the total sulphur and sulphate ion contents as well as thiol levels of the *old3 *mutant and their respective wild types. Total sulphur and sulphate ion content was increased while the thiol content was lower in *old3-1 *and *old3-2/oasa1.1 *lines as compared to their wild types. The data show that the *OAS-A1/OLD3*, independently from the *odd-ler *gene affects sulphur balance as well as thiol levels.

### *old3 *mutant lines show an increased sensitivity to cadmium stress

*OAS-A1/OLD3 *gene expression is up-regulated in response to cadmium-induced stress and this may allow for enhanced cadmium tolerance [36]. The effect of cadmium on seedling growth of the *old3-1 *and *old3-2/oasa1.1 *mutants and their wild types were investigated. Figure 7c shows the effect of various cadmium concentrations on plant growth. At 150 μM cadmium, all tested genotypes showed a considerable decrease in fresh weight as compared to growth without cadmium. In contrast, the *old3-1 *and *old3-2/oas-a1.1 *lines showed significant reductions in fresh weight already from 50 μM cadmium and onwards. Thus, *OAS-A1/OLD3 *is involved in cadmium tolerance and the *old3-1 *mutation increases the cadmium sensitivity, independent of the presence of the *odd-ler *or *odd-col *gene.

## Discussion

Cysteine is synthesised in the cytosol, the plastids and mitochondria. The *Arabidopsis *genome contains OAS-TL isoforms for each of the different cellular locations. Recently the effects of T-DNA insertions in the genes encoding the different isoforms were studied [18]. Although the cytosolic OAS-A1/OLD3 isoform contributes to 44% of total OAS-TL activity in leaf and ~80% in root, it was reported that the OAS-A1/OLD3 isoform is completely dispensable [18]. However, others found that loss-of function cytosolic *OAS-TL *mutants resulted in compromised antioxidant capacity of the cytosol [17]. Here we report that a single amino-acid change in OAS-A1/OLD3 compromises OAS-TL activity and uncovers a novel role of this enzyme in cell death signalling.

The semi-dominant *old3-1 *mutation causes a Gly^162 ^to Glu^162 ^substitution in OAS-A1/OLD3. The *old3-1 *mutation results in a dysfunctional enzyme *in vitro *and likely *in vivo *as well. The 3D structure of the OAS-A1/OLD3 has been resolved [28] and this allows for an explanation for the compromised enzyme activity: the small and neutral Gly residue is substituted with a much larger charged amino acid Glu that is in close proximity to the sixth α-helix, therefore likely causing a positional movement in the α-helices 5 and 6, and subsequent shifts of the associated residues in these helices. Consequently, residues involved in the positioning of the substrate and the pyridoxal 5'-phosphate (PLP) cofactor, which is ~16 A away from Gly^162^, might be affected by these helical shifts [37,28].

The *old3-1 *early leaf death phenotype is a result of the activity of the mutated *old3-1 *allele, rather than the absence of the wild type *OAS-A1/OLD3 *allele, in combination with a natural variant gene *odd-ler*. The phenotype therefore depends on the genomic context. The *odd-ler-*like alleles are present in the minority of accessions tested and the presence of *odd-col/odd-ler *alleles does not correlate with the geographic origins of the accessions, or with their phylogeny relationships as illustrated with molecular markers [38]. It is unclear how the mutated OAS-TL may cause cell death in combination with the *odd-ler *gene. The CSC formation and protein-protein interaction of its subunits play essential regulatory roles in plants [1] and the old3-1 mutant protein may affect the formation of the CSC and SAT activity. A changed OAS-TL conformation could exert a dominant-negative effect in combination with the *odd-ler *gene product. The leaf death phenotype moreover strictly depends on temperature and can conveniently be switched on and off by changing the growth conditions. It seems possible that a higher temperature may alter the folding of the mutated protein or abolish the interaction with the *odd-ler *gene product or a protein that is involved in the leaf death phenotype. Formally it cannot be excluded that the absence of cytosolic OAS-TL activity causes the early leaf death phenotype in combination with the *odd-ler *allele. In this scenario, the early leaf death phenotype observed in the L*er*-0 plants transformed with the *old3-1 *gene would have to be a result of co-suppression of the wild type *OLD3 *gene by the transgene. However, since the obtained transformants all had the early leaf death phenotype and no wild type transformants were found, this possibility seems unlikely.

The *old3-2 *mutant used in this study is identical to the *oas-a1.1 *mutation and is likely a knock-out allele [17]. Similar to what has been found earlier [17,18], the *old3-2/oas-a1.1 *mutation or the *old3-1 *mutation in combination with the homozygous *odd-col *allele (*old3-1old3-1odd-colodd-col*) resulted in plants that were indistinguishable from the wild type, when grown under standard conditions. Therefore, the compromised activity of OAS-A1/OLD3 in *old3-2 *plants does not induce the cell death phenotype. However, a significant increase in both total sulphur and sulphate ion was found in the *old3-2/oas-a1.1 *mutant. Similar differences were not found by Heeg et al. [18], perhaps due to differences in growth conditions and/or plant age. In addition, a decrease in non-protein thiol contents was found in the *old3-1 and old3-2/oas-a1.1 *mutant lines. GSH is a non-protein thiol and others [17] found a reduction in GSH levels in *old3-2/oas-a1.1 *plants. These results suggest that the compromised OAS-A1/OLD3 activity in the mutant causes reduced sulphate assimilation and lower organic sulphur levels. However, the changes are small and may have little, if any, impact on plant growth when grown under standard growth conditions [18,17].

Plants have developed defence systems to cope with environmental stresses such as heavy metal pollution. Cysteine is required for the synthesis of GSH and is as such involved in plant responses to toxic levels of heavy metals [21]. *OAS-A1/OLD3 *is known to be involved in the defence response of *Arabidopsis *against abiotic stresses such as salinity and the presence of heavy metals [17,39,40]. Moreover, its higher expression resulted in increased cadmium tolerance [39,36]. Thus, increased OAS-A1/OLD3 activity enhances cadmium resistance. Here we show that plants with compromised OAS-A1/OLD3 function are more sensitive to cadmium stress. Consequently, the OAS-A1/OLD3 is required for coping with cadmium-induced stress and the mitochondrial and/or the plastidic OAS-TL cannot fully compensate for the lack of OAS-A1/OLD3. The function of the OAS-A1/OLD3 may therefore be to quickly respond to environmental stress, while its function during growth under environmentally ideal conditions may be limited. Consistent with our results, *old3-2/oas-a1.1 *seedlings exhibited an enhanced sensitivity to cadmium-induced stress [17]. In addition, the data from transcriptomic analysis of *old3-2/oas-a1.1 *mutant plants grown under non-stressed conditions suggested the involvement of *OAS-A1/OLD3 *in plant responses to stress, and in particular, to ROS-induced stress. These findings coincide with phenotypes observed in leaves of *old3-2/oas-a1.1 *mutants, including histochemical detection of H_2_O_2 _as well as lesions characteristic for cell death [17]. Moreover, a recent research showed the existence of OAS-A1/OLD3 in the peroxisomes, which are actively involved in ROS detoxification in leaves [41]. Thus, the results presented here are consistent with the suggestion that OAS-A1/OLD3 deficiency results in a constitutively reduced capacity to eliminate cytosolic ROS [17]. Moreover the reported phenotype of the *oas-a1.1 *mutant shows some resemblance with the *old3-1 *early leaf death phenotype. *oas-a1.1 *mutant plants have microscopic lesions and the massive cell death observed in *old3-1 *plants may be the result of the same phenomenon on a much larger scale. The leaf death phenotype coincides with increased expression of the general oxidative stress-related marker *DEFL *and with other marker genes commonly associated with the activation of the plant defence response. It seems appealing to suggest that the presence of *odd-ler *may amplify the effect of OAS-A1 deficiency on ROS elimination in *old3-1 *mutant plants. However, the leaf death phenotype requires the presence of the mutated *old3-1*/*OAS-A1 *gene, rather than the absence of the wild type one. The question remains how the mutated protein causes the phenotype. It seems inevitable that the Gly^162 ^to Glu^162 ^substitution causes a conformational change and this may alter the interaction with SAT. It is notable that knockout of four of the 5 SATs causes dwarfism, depending on the SAT remaining [10]. The early leaf death may be an extreme manifestation of the observed dwarfism as a result of an *odd-ler*-dependent changed interaction between SAT and old3-1. However, an altered conformation could equally well change the function or amplify an otherwise minor role of the protein.

## Conclusions

This study describes an EMS-induced mutation in the cytosolic OAS-TL. The mutation results in a protein that has no *in vitro *OAS-TL activity anymore, resulting in altered sulphur balance and cadmium hypersensitivity. The mutated gene furthermore causes a temperature-dependent lethal phenotype in combination with the genome-dependent gene *odd-ler*. The nature of the *odd-ler/old3-1 *interaction is still unclear, but the identification of the *odd-ler *gene, together with analysis of the leaf death syndrome will allow us to unravel this unique cell death pathway.

## Methods

### Plant material and growth conditions

*Arabidopsis thaliana *accessions Landsberg *erecta *(L*er*-0) and Colombia (Col-0) were mainly used in this study. Several other accessions were obtained from the NASC (Nottingham *Arabidopsis *Stock Centre). The *old3-1 *(*old3-1old3-1odd-ler odd-ler*) mutant has been described elsewhere [24]. *old3-1old3-1odd-colodd-col *genotype was selected from an F_2 _population generated from a cross between *old3-1OLD3odd-lerodd-ler *with Col-0 (*OLD3OLD3odd-colodd-col)*. The *old3-1old3-1odd-colodd-col *plants therefore carry homozygous L*er*-0 DNA around the *old3-1 *gene and homozygous Col-0 DNA around the *odd-col *locus, as confirmed with DNA markers. The rest of the genome is mixed Col-0/L*er*-0. SALK line 072213, carrying a T-DNA insertion in the *OAS-A1/OLD3 *gene in Col-0 background was obtained from NASC under number N572213 [42]. A plant line homozygous for the insertion was designated as *old3-2*. The T-DNA insertion was detected with a PCR product from the primer pair PrRuG659: TGGTTCACGTAGTGGGCCATCG/PrRuG761: TACACCAATGGAGTGTTCCCAATCA. Surface-sterilized seeds of L*er*-0, Col-0, *old3-1 *and *old3-2 *genotypes were sown on MS media [43], pH 5.6, 1% sucrose, 1% agar, and CdCl_2 _at various concentrations. Seeds were kept at 4°C for 3 days and subsequently transferred to a growth chamber (21°C, 16 h light/8 h dark) for 16 days before harvesting. Soil-grown plants were sown on an organic-rich soil (TULIP PROFI No.4, BOGRO B.V., Hardenberg, The Netherlands) as described [24].

### Map-based cloning and plant transformation

To identify the *old3-1 *mutation, approximately 5000 homozygous *old3-1 *(*old3-1old3-1odd-lerodd-ler*) F_2 _seedlings with a lethal phenotype were selected from a mapping population generated by crossing heterozygous *old3-1 *(*old3-1OLD3odd-ler odd-ler*) plants with Col-0 (*OLD3OLD3odd-colodd-col*). DNA was isolated using the SHORTY quick preparation method (http://preuss.bsd.uchicago.edu/protocols/Plantdna.html). The *old3-1 *mutation was mapped to the *Arabidopsis *genome using CAPS, SSLP and SNP markers. Potential SNPs [44], were selected and primers that specifically amplified Col-0 DNA fragments were designed using the Web SNAPER program [45]. The mutation was mapped onto a 14 kb region spanning 8 open reading frames. Sequence analyses revealed a single nucleotide change inside *OAS-A1/OLD3*. Oligonucleotide primers PrRuG760 (CTATGATCCTTCCGGTGGTGAGAA) and PrRuG775 (GATGGAAGCAAAGACGCAATGTAACTAA) were used to amplify the mutated *OAS-A1 *gene from *old3-1 *plants. The genomic fragment was cloned into pGreen0229 and *Agrobacterium*-mediated transformation [46,47] of the construct into the wild type L*er*-0 was performed to confirm that the *old3-1 *mutation causes the early leaf death phenotype. In order to distinguish Ho-*old3-1 *(ie, *old3-1old3-1*) and Het-*old3-1 *(ie, *OLD3old3-1*) plants, the primers PrRuG760 (CTATGATCCTTCCGGTGGTGAGAA) and PrRuG775 (GATGGAAGCAAAGACGCAATGTAACTAA) were employed to amplify the *OAS-A1 *gene from L*er*-0, *old3-1old3-1odd-lerodd-ler *and *old3-1OLD3odd-lerodd-ler *plants. The obtained 2928 bp PCR product was digested by *Sau*96I to visualise the polymorphism between the *OLD3 *and *old3-1 *alleles. The *odd-ler *gene was mapped at the lower arm of chromosome 3, approximately 4 centiMorgans south of SSLP marker K11J14 [24].

### Cloning, enzyme activity assay and complementation of ΔCys *E.coli*

The cytosolic forms of *OAS-A1/OLD3 *and *old3-1 *were amplified from L*er*-0 and *old3-1 *cDNAs, respectively, using specific primers as described [28]. The PCR products were sub-cloned into a pGEM-T Easy vector (Promega) and sequenced. The cDNA fragments were cut with NdeI and BamHI and ligated into NcoI/BamHI digested pTrc99A [48] and pET15b (Novagen) to create the pTrc99A:*OLD3*, pTrc99A:*old3-1*, pET15b:*OLD3 *and pET15b:*old3-1 *constructs.

OAS-A1/OLD3 and old3-1 protein was purified from *E. coli *BL21 (DE3) cells transformed with pET15b:*OLD3 *and pET15b:*old3-1 *constructs as described elsewhere [28]. Transformed BL21 (DE3) cells were grown on LB at 37°C until A600 ~0.8 and protein expression was induced with 1 mM 1-thio-*β*-D-galactopyranoside for 4 h at 37°C. Bound proteins were eluted from Ni^2+^-NTA agarose columns with buffer containing 150 mM imidazole. Eluted proteins were dialyzed against 100 mM Mopso (pH 7.0) and stored at -20°C. OAS-A1 enzyme activity was determined as described [28] using the Gaitonde method [30]. Briefly, the reaction was started with the addition of 26.7 ng (3.1 nM) of OAS-A1/OLD3 and old3-1 protein and incubated for 5, 10 and 15 min and subsequently stopped by adding 50 μl trichloroacetic acid. After centrifugation, 0.25 ml supernatant was added to 0.25 ml acid-ninhydrin reagent, and placed in a boiling water bath for 5 min. After cooling, 0.5 ml 100% cold ethanol was added and the extinction at 560 nm was measured. The standard curve for cysteine was made as described [49].

For *E. coli *NK3 (*ΔtrpE5 leu-6 hsdR hsdM+ cysK cysM*) [29] complementation, *E. coli *cells were transformed with the empty vector pTrc99A, pTrc99A:*OLD3 *and pTrc99A:*old3-1 *and grown on 5× M9 Minimal Media-containing 1.5% agar plates, supplemented with 0.2 gL^-1 ^leucine and tryptophan, 50 μgml^-1 ^ampiciline, and 0.5 mM cysteine where indicated.

### Physiological analyses

Chlorophyll content and ion leakage was measured as described [24]. To measure total sulphur, sulphate ion and thiols, plants were grown in soil saturated with 25% Hoagland solution for 27 days, at 25 °C and 70% relative humidity, and a 16 hr/8 hr light/dark cycle (200 μmolm^-2^s^-1^). The above ground parts of plants were harvested for fresh weight and dry weight measurement. Dry plant materials were used for total sulphur measurement using the barium sulphate precipitation method [50] and for anion measurement using HPLC-based methods [51]. Parts of the fresh samples were used for the measurement of total water-soluble non-protein sulfhydryl (SH) compounds following the method described [52]. For the total sulphur and sulphate ion content data of *old3-1old3-1odd-lerodd-ler *mutants, one replicate of 50 plants was used for analysis due to the small size of plants and for other data points four replicates of 17 plants were used.

### RNA isolation and Real-Time PCR

Total RNA was isolated and purified using the Qiagen RNeasy plant mini kit according to the manufacturer's protocol. Fifteen hundred nanograms of RNA were used as template for first-strand cDNA synthesis using 200 U of RevertAid H-minus MMuLV reverse transcriptase (Fermentas USA) and an oligo (dT) primer. Primer pairs for Real-Time PCR were designed using the open-source PCR primer design program PerlPrimer v1.1.10 [53]. Applied Biosystems 7300 Real-Time PCR system and the Applied Biosystems power SYBR Green PCR Master Mix kit was used for the Real-Time PCR amplification according to the manufacturer's protocol. Oligonucleotide primers PrRuG2443 (CTTAGTCATTTCCGATGTGCC) and PrRuG2444 (GCATCTTCCACCTTTAGCTC) were used to amplify *DEFL *(*AT2G43510*) and primers PrRuG1699 (TCTCCGCTTTGAATTGTCTC) and PrRuG1700 (TATGAGCTTGGAAAGAAAGAGC) were used to amplify *ACTIN2 *(*AT3G18780*). The Real-Time PCR program was 2 min at 94°C, 40× (94°C for 10 sec/58°C for 10 sec/72°C for 25 sec). The PCR reaction was followed by a meltcurve analysis to confirm that a single band was amplified and no primer dimers were present. The obtained data was analysed with Bio-Rad software.

## Authors' contributions

RK and HCJ did the physiological and gene expression analysis. HCJ and JS identified the *old3-1 *mutation. RK did the structure analysis. RK and GE determined the OAS-TL activities. JH participated in the design and coordination of the study. PPD conceived of the study, participated in the design and coordination the work. RK, HCJ and PD drafted the manuscript. All authors contributed to the draft and read and approved the final manuscript.

## References

[B1] Wirtz M Hell R Dominant-negative modification reveals the regulatory function of the multimeric cysteine synthase protein complex in transgenic tobacco Plant Cell 2007 19 2 625 639 10.1105/tpc.106.043125 17293569 PMC1867341

[B2] May MJ Vernoux T Leaver C Van Montagu M Inze D Glutathione homeostasis in plants: implications for environmental sensing and plant development Journal of Experimental Botany 1998 49 649 667 10.1093/jexbot/49.321.649

[B3] Buchner P Takahashi H Hawkesford MJ Plant sulphate transporters: co-ordination of uptake, intracellular and long-distance transport J Exp Bot 2004 55 404 1765 1773 10.1093/jxb/erh206 15258169

[B4] Leustek T Martin MN Bick J-A Davies JP PATHWAYS AND REGULATION OF SULFUR METABOLISM REVEALED THROUGH MOLECULAR AND GENETIC STUDIES Annu Rev Plant Physiol Plant Mol Biol 2000 51 141 165 10.1146/annurev.arplant.51.1.141 15012189

[B5] Saito K Regulation of sulfate transport and synthesis of sulfur-containing amino acids Curr Opin Plant Biol 2000 3 3 188 195 10837270

[B6] Wirtz M Droux M Hell R O-acetylserine (thiol) lyase: an enigmatic enzyme of plant cysteine biosynthesis revisited in Arabidopsis thaliana J Exp Bot 2004 55 404 1785 1798 10.1093/jxb/erh201 15258168

[B7] Droux M Ruffet ML Douce R Job D Interactions between serine acetyltransferase and O-acetylserine (thiol) lyase in higher plants--structural and kinetic properties of the free and bound enzymes Eur J Biochem 1998 255 1 235 245 10.1046/j.1432-1327.1998.2550235.x 9692924

[B8] Feldman-Salit A Wirtz M Hell R Wade RC A mechanistic model of the cysteine synthase complex J Mol Biol 2009 386 1 37 59 10.1016/j.jmb.2008.08.075 18801369

[B9] Wirtz M Hell R Functional analysis of the cysteine synthase protein complex from plants: structural, biochemical and regulatory properties J Plant Physiol 2006 163 3 273 286 10.1016/j.jplph.2005.11.013 16386330

[B10] Watanabe M Mochida K Kato T Tabata S Yoshimoto N Noji M Saito K Comparative Genomics and Reverse Genetics Analysis Reveal Indispensable Functions of the Serine Acetyltransferase Gene Family in Arabidopsis Plant Cell 2008 20 9 2484 2496 10.1105/tpc.108.060335 18776059 PMC2570737

[B11] Zimmermann P Hirsch-Hoffmann M Hennig L Gruissem W GENEVESTIGATOR. Arabidopsis microarray database and analysis toolbox Plant Physiol 2004 136 1 2621 2632 10.1104/pp.104.046367 15375207 PMC523327

[B12] Winter D Vinegar B Nahal H Ammar R Wilson GV Provart NJ An "electronic fluorescent pictograph" browser for exploring and analyzing large-scale biological data sets PLoS One 2007 2 1 e718 10.1371/journal.pone.0000718 17684564 PMC1934936

[B13] Hell R Bork C Bogdanova N Frolov I Hauschild R Isolation and characterization of two cDNAs encoding for compartment specific isoforms of O-acetylserine (thiol) lyase from Arabidopsis thaliana FEBS Lett 1994 351 2 257 262 10.1016/0014-5793(94)00872-8 8082776

[B14] Hesse H Lipke J Altmann T Hofgen R Molecular cloning and expression analyses of mitochondrial and plastidic isoforms of cysteine synthase (O-acetylserine(thiol)lyase) from Arabidopsis thaliana Amino Acids 1999 16 2 113 131 10.1007/BF01321531 10319184

[B15] Hatzfeld Y Maruyama A Schmidt A Noji M Ishizawa K Saito K beta-Cyanoalanine synthase is a mitochondrial cysteine synthase-like protein in spinach and Arabidopsis Plant Physiol 2000 123 3 1163 1171 10.1104/pp.123.3.1163 10889265 PMC59079

[B16] Yamaguchi Y Nakamura T Kusano T Sano H Three Arabidopsis genes encoding proteins with differential activities for cysteine synthase and beta-cyanoalanine synthase Plant Cell Physiol 2000 41 4 465 476 10845460 10.1093/pcp/41.4.465

[B17] Lopez-Martin MC Becana M Romero LC Gotor C Knocking out cytosolic cysteine synthesis compromises the antioxidant capacity of the cytosol to maintain discrete concentrations of hydrogen peroxide in Arabidopsis Plant Physiol 2008 147 2 562 572 10.1104/pp.108.117408 18441224 PMC2409041

[B18] Heeg C Kruse C Jost R Gutensohn M Ruppert T Wirtz M Hell R Analysis of the Arabidopsis O-acetylserine(thiol)lyase gene family demonstrates compartment-specific differences in the regulation of cysteine synthesis Plant Cell 2008 20 1 168 185 10.1105/tpc.107.056747 18223034 PMC2254930

[B19] Noctor G Strohm M Jouanin L Kunert KJ Foyer CH Rennenberg H Synthesis of Glutathione in Leaves of Transgenic Poplar Overexpressing [gamma]-Glutamylcysteine Synthetase Plant Physiol 1996 112 3 1071 1078 12226433 10.1104/pp.112.3.1071 PMC158033

[B20] Strohm M Jouanin L Kunert KJ Pruvost C Polle A Foyer CH Rennenberg H REGULATION OF GLUTATHIONE SYNTHESIS IN LEAVES OF TRANSGENIC POPLAR (POPULUS-TREMULA X POPULUS-ALBA) OVEREXPRESSING GLUTATHIONE SYNTHETASE Plant Journal 1995 7 1 141 145 10.1046/j.1365-313X.1995.07010141.x

[B21] Cobbett CS Phytochelatins and their roles in heavy metal detoxification Plant Physiol 2000 123 3 825 832 10.1104/pp.123.3.825 10889232 PMC1539264

[B22] Meyer AJ Hell R Glutathione homeostasis and redox-regulation by sulfhydryl groups Photosynth Res 2005 86 3 435 457 10.1007/s11120-005-8425-1 16315075

[B23] Mullineaux PM Rausch T Glutathione, photosynthesis and the redox regulation of stress-responsive gene expression Photosynth Res 2005 86 3 459 474 10.1007/s11120-005-8811-8 16328783

[B24] Jing H-C Sturre MJG Hille J Dijkwel PP Arabidopsis onset of leaf death mutants identify a regulatory pathway controlling leaf senescence Plant J 2002 32 1 51 63 10.1046/j.1365-313X.2002.01400.x 12366800

[B25] Brodersen P Petersen M Pike HM Olszak B Skov S Ødum N Jørgensen LB Brown RE Mundy J Knockout of Arabidopsis ACCELERATED-CELL-DEATH11 encoding a sphingosine transfer protein causes activation of programmed cell death and defense Genes & Development 2002 16 4 490 502 10.1101/gad.218202 11850411 PMC155338

[B26] Gadjev I Vanderauwera S Gechev TS Laloi C Minkov IN Shulaev V Apel K Inze D Mittler R Van Breusegem F Transcriptomic footprints disclose specificity of reactive oxygen species signaling in Arabidopsis Plant Physiol 2006 141 2 436 445 10.1104/pp.106.078717 16603662 PMC1475436

[B27] Bogdanova N Hell R Cysteine synthesis in plants: protein-protein interactions of serine acetyltransferase from Arabidopsis thaliana Plant J 1997 11 2 251 262 10.1046/j.1365-313X.1997.11020251.x 9076992

[B28] Bonner ER Cahoon RE Knapke SM Jez JM Molecular basis of cysteine biosynthesis in plants: structural and functional analysis of O-acetylserine sulfhydrylase from Arabidopsis thaliana J Biol Chem 2005 280 46 38803 38813 10.1074/jbc.M505313200 16166087

[B29] Ploeg JR van der Barone M Leisinger T Functional analysis of the Bacillus subtilis cysK and cysJI genes FEMS Microbiol Lett 2001 201 1 29 35 10.1111/j.1574-6968.2001.tb10728.x 11445163

[B30] Gaitonde MK A spectrophotometric method for the direct determination of cysteine in the presence of other naturally occurring amino acids Biochem J 1967 104 2 627 633 6048802 10.1042/bj1040627 PMC1270629

[B31] Burandt P Schmidt A Papenbrock J Three O -acetyl-L-serine(thiol)lyase isoenzymes from Arabidopsis catalyse cysteine synthesis and cysteine desulfuration at different pH values Journal of Plant Physiology 2002 159 2 111 119 10.1078/0176-1617-00611

[B32] Jost R Berkowitz O Wirtz M Hopkins L Hawkesford MJ Hell R Genomic and functional characterization of the oas gene family encoding O-acetylserine (thiol) lyases, enzymes catalyzing the final step in cysteine biosynthesis in Arabidopsis thaliana Gene 2000 253 2 237 247 10.1016/S0378-1119(00)00261-4 10940562

[B33] Hesse H Nikiforova V Gakiere B Hoefgen R Molecular analysis and control of cysteine biosynthesis: integration of nitrogen and sulphur metabolism Journal of Experimental Botany 2004 55 401 1283 1292 10.1093/jxb/erh136 15133050

[B34] Howarth JR Dominguez-Solis JR Gutierrez-Alcala G Wray JL Romero LC Gotor C The serine acetyltransferase gene family in Arabidopsis thaliana and the regulation of its expression by cadmium Plant Mol Biol 2003 51 4 589 598 10.1023/A:1022349623951 12650624

[B35] Rauser WE Metal-Binding Peptides in Plants Sulfur Nutrition and Assimilation in Higher Plants 1993 239 251

[B36] Dominguez-Solis JR Lopez-Martin MC Ager FJ Ynsa MD Romero LC Gotor C Increased cysteine availability is essential for cadmium tolerance and accumulation in Arabidopsis thaliana Plant Biotechnol J 2004 2 6 469 476 10.1111/j.1467-7652.2004.00092.x 17147619

[B37] Francois JA Kumaran S Jez JM Structural basis for interaction of O-acetylserine sulfhydrylase and serine acetyltransferase in the Arabidopsis cysteine synthase complex Plant Cell 2006 18 12 3647 3655 10.1105/tpc.106.047316 17194764 PMC1785398

[B38] Barth S Melchinger AE Lubberstedt T Genetic diversity in Arabidopsis thaliana L. Heynh. investigated by cleaved amplified polymorphic sequence (CAPS) and inter-simple sequence repeat (ISSR) markers Mol Ecol 2002 11 3 495 505 10.1046/j.0962-1083.2002.01466.x 11918784

[B39] Dominguez-Solis JR Gutierrez-Alcala G Vega JM Romero LC Gotor C The cytosolic O-acetylserine(thiol)lyase gene is regulated by heavy metals and can function in cadmium tolerance J Biol Chem 2001 276 12 9297 9302 10.1074/jbc.M009574200 11121418

[B40] Barroso C Romero LC Cejudo FJ Vega JM Gotor C Salt-specific regulation of the cytosolic O-acetylserine(thiol)lyase gene from Arabidopsis thaliana is dependent on abscisic acid Plant Mol Biol 1999 40 4 729 736 10.1023/A:1006285016296 10480396

[B41] Reumann S Babujee L Ma C Wienkoop S Siemsen T Antonicelli GE Rasche N Luder F Weckwerth W Jahn O Proteome analysis of Arabidopsis leaf peroxisomes reveals novel targeting peptides, metabolic pathways, and defense mechanisms Plant Cell 2007 19 10 3170 3193 10.1105/tpc.107.050989 17951448 PMC2174697

[B42] Alonso JM Stepanova AN Leisse TJ Kim CJ Chen H Shinn P Stevenson DK Zimmerman J Barajas P Cheuk R Genome-wide insertional mutagenesis of Arabidopsis thaliana Science 2003 301 5633 653 657 10.1126/science.1086391 12893945

[B43] Murashige T Skoog F A Revised Medium for Rapid Growth and Bio Assays with Tobacco Tissue Cultures Physiologia Plantarum 1962 15 3 473 497 10.1111/j.1399-3054.1962.tb08052.x

[B44] Jander G Norris SR Rounsley SD Bush DF Levin IM Last RL Arabidopsis map-based cloning in the post-genome era Plant Physiol 2002 129 2 440 450 10.1104/pp.003533 12068090 PMC1540230

[B45] Drenkard E Richter BG Rozen S Stutius LM Angell NA Mindrinos M Cho RJ Oefner PJ Davis RW Ausubel FM A simple procedure for the analysis of single nucleotide polymorphisms facilitates map-based cloning in Arabidopsis Plant Physiol 2000 124 4 1483 1492 10.1104/pp.124.4.1483 11115864 PMC1539302

[B46] Hellens RP Edwards EA Leyland NR Bean S Mullineaux PM pGreen: a versatile and flexible binary Ti vector for Agrobacterium-mediated plant transformation Plant Mol Biol 2000 42 6 819 832 10.1023/A:1006496308160 10890530

[B47] Clough SJ Bent AF Floral dip: a simplified method for Agrobacterium-mediated transformation of Arabidopsis thaliana Plant J 1998 16 6 735 743 10.1046/j.1365-313x.1998.00343.x 10069079

[B48] Amann E Ochs B Abel KJ Tightly regulated tac promoter vectors useful for the expression of unfused and fused proteins in Escherichia coli Gene 1988 69 2 301 315 10.1016/0378-1119(88)90440-4 3069586

[B49] Nakamura K Hayama A Masada M Fukushima K Tamura G Measurement Of Serine Acetyltransferase Activity in Crude Plant-Extracts By a Coupled Assay System Using Cysteine Synthase Plant and Cell Physiology 1987 28 5 885 891

[B50] Jones JB Jr Determining total sulphur in plant tissue using the HACH kit spectrophotometer technique Sulphur in Agriculture 1995 19 58 62

[B51] Maas FM Hoffmann I Vanharmelen MJ Dekok LJ Refractometric Determination Of Sulfate And Other Anions in Plants Separated By High-Performance Liquid-Chromatography Plant and Soil 1986 91 1 129 132 10.1007/BF02181825

[B52] Dekok LJ Graham M Levels Of Pigments, Soluble-Proteins, Amino-Acids And Sulfhydryl Compounds in Foliar Tissue Of Arabidopsis-Thaliana During Dark-Induced And Natural Senescence Plant Physiology and Biochemistry 1989 27 2 203 209

[B53] Marshall OJ PerlPrimer: cross-platform, graphical primer design for standard, bisulphite and real-time PCR Bioinformatics 2004 20 15 2471 2472 10.1093/bioinformatics/bth254 15073005

